# High frame-rate resolution of cell division during *Candida albicans* filamentation

**DOI:** 10.1016/j.fgb.2016.02.001

**Published:** 2016-03

**Authors:** Darren D. Thomson, Judith Berman, Alexandra C. Brand

**Affiliations:** a School of Medicine, Medical Sciences & Nutrition, University of Aberdeen, Foresterhill, Aberdeen AB25 2ZD, UK; b Manchester Fungal Infection Group, Institute of Inflammation and Repair, University of Manchester, CTF Building, Grafton Street, Manchester M13 9NT, UK; c Department of Microbiology and Biotechnology, George S. Wise Faculty of Life Sciences, Tel Aviv University, Ramat Aviv 69978, Israel

**Keywords:** GFP, green fluorescent protein, YFP, yellow fluorescent protein, DIC, Differential Interference Contrast, Nuclear division, Hypha, Septum

## Abstract

The commensal yeast, *Candida albicans*, is an opportunistic pathogen in humans and forms filaments called hyphae and pseudohyphae, in which cell division requires precise temporal and spatial control to produce mononuclear cell compartments. High-frame-rate live-cell imaging (1 frame/min) revealed that nuclear division did not occur across the septal plane. We detected the presence of nucleolar fragments that may be extrachromosomal molecules carrying the ribosomal RNA genes. Cells occasionally maintained multiple nucleoli, suggesting either polyploidy, multiple nuclei and/or aneuploidy of ChrR., while the migration pattern of sister nuclei differed between unbranched and branched hyphae. The presented movie challenges and extends previous concepts of *C. albicans* cell division.

## Highlights

• Nuclear divisions occur within the mother compartment and not across the septal plane.
• Maintenance of >1 nucleolar signal in some cells suggested altered ploidy or aneuploidy.
• The appearance of small Nop1 fragments suggested the generation of extrachromosomal rDNA circles.
• The migration pattern of sister nuclei differed between unbranched and branched hyphae.

## 1 Introduction

*Candida albicans* is a multimorphic fungus that lives as a commensal yeast in humans and produces invasive hyphae and pseudohyphae as an opportunistic pathogen in patient groups with underlying immune deficiencies. Pseudohyphae are elongated yeast cells that fail to undergo cell separation following cytokinesis, resulting in chains of cells with constrictions at the septa that form at the mother-bud evagination site. In contrast, septin rings in hyphae are deposited from the growing tip as it passes the future site of septation (the presumptum) (Sudbery, 2001). In addition, the Spitzenkörper, an apical ‘body’ of vesicles, appears as a bright Mlc1-GFP (Myosin light-chain 1) spot at the hyphal tip throughout the cell cycle (Crampin et al., 2005). Another distinctive feature of hyphae is the lack of constrictions in the cell wall. Although different culture conditions can enrich for one form over the other, the yeast and hyphal morphologies comprise two ends of a continuum and fungal lesions in infected solid organ tissues invariably contain both cell types (Merson-Davies and Odds, 1989).

In contrast to most multinuclear filamentous fungi that undergo organellar streaming through open septal pores, *C. albicans* hyphae are compartmentalized by septa containing a 25 nm micropore that inhibits such traffic (Gow et al., 1980). *C. albicans* therefore requires tight regulation of nuclear division to ensure that each daughter cell contains a single nucleus prior to septum closure. Nonetheless, unusual mitotic divisions can give rise to *C. albicans* tetraploids, aneuploids or haploids (Suzuki et al., 1986, Rustchenko-Bulgac and Howard, 1993, Hickman et al., 2013), especially after drug exposure (Selmecki et al., 2006, Harrison et al., 2014).

Here we used high frame-rate time-lapse microscopy to report new insights into the spatio-temporal sequence of cell-cycle events in wild-type *C. albicans* cells that challenge our previous understanding of this process.

## 2 Results and discussion

The movie presented shows the growth of constitutively polarized *C. albicans* cells after initial evagination of a germ tube from 4 mother yeast cells (Yeast Cells 1–4, Fig. 2A, Movie 1). Filamentous growth was induced in serum at 37 °C in a micro-fabricated chamber featuring parallel walls (Brand et al., 2008). Nucleoli were visualized by expression of ribonucleolar protein, Nop1, fused to YFP and were used as a proxy for nuclear localization, as shown by co-localization of Nop1 with histones inside the nuclear membrane in *C. albicans* hyphae (Finley and Berman, 2005). Septum formation was visualized using Cdc3, one of the 5 *C.* *albicans* septins that comprise the septin ring, fused to GFP. The contrasting spatial organization of the two marker proteins rendered them easily distinguishable in the same fluorescence channel (Finley and Berman, 2005).

### 2.1 Sequence of cell division events

**Fig. 1:**
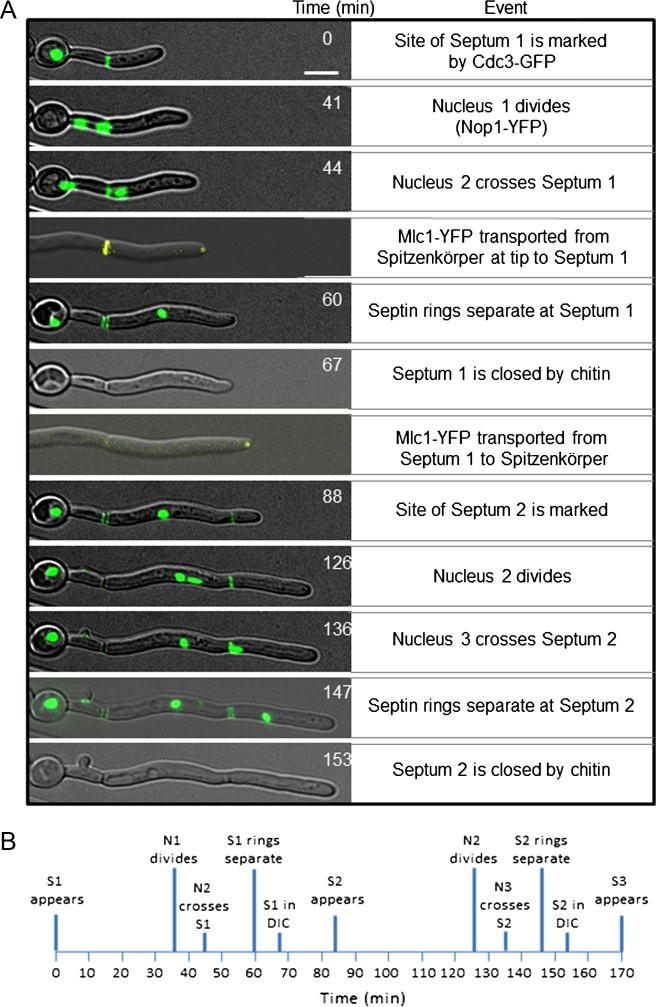
Temporal and spatial sequence of cell-division events during polarized growth in *Candida albicans*. (A) The nucleolus and septin rings were visualized in double-tagged strain 8860 expressing Nop1-YFP and Cdc3-GFP as markers, respectively. In a separate strain, the Spitzenkörper was visualized using Mlc1-YFP. The timing of cell-cycle events was normalized across strains using the appearance of the closed septum, visible in DIC in both strains, as a shared reference point. Frame rate = 1 frame/min, bar = 5 μm. (B) Time course of cell-division events (194 events observed) (S, septum; N, nucleus; DIC, Differential Interference Contrast).

During early hyphal growth, the Cdc3-GFP septins were observable as a spot within the hyphal tip until they marked the position of the first nascent septum by forming a single polymeric ring at the internal face of the plasma membrane. After mitosis and arrival of a sister nucleus in the daughter cell, the Cdc3-GFP signal at the presumptum separated into two distinguishable rings to make way for chitin deposition and formation of the septal wall. Closure of the septum by chitin deposition was observed using DIC microscopy. During septin ring separation, the Mlc1-YFP signal at the Spitzenkörper dimmed temporarily as a sub-population of Mlc1-YFP was seen streaming away from the Spitzenkörper to concentrate at the septum (Fig. 1A). After septal closure, the Mlc1-YFP signal at the septum was lost and the fluorescent signal returned to its previous intensity at the Spitzenkörper (Thomson et al., 2014). The observation that a subpopulation of Mlc1 can be temporarily relocalized to the septum suggests that the protein copy number within apical cells is relatively stable during growth, consistent with the idea that *C.* *albicans* hyphae grow using a constant volume of cytoplasm pushed forwards from the previous cell (Gow and Gooday, 1982). The sequence of cell division events and their relative timings are illustrated in Fig. 1A and B.

### 2.2 Nuclear division, mobility and ploidy

**Fig. 2:**
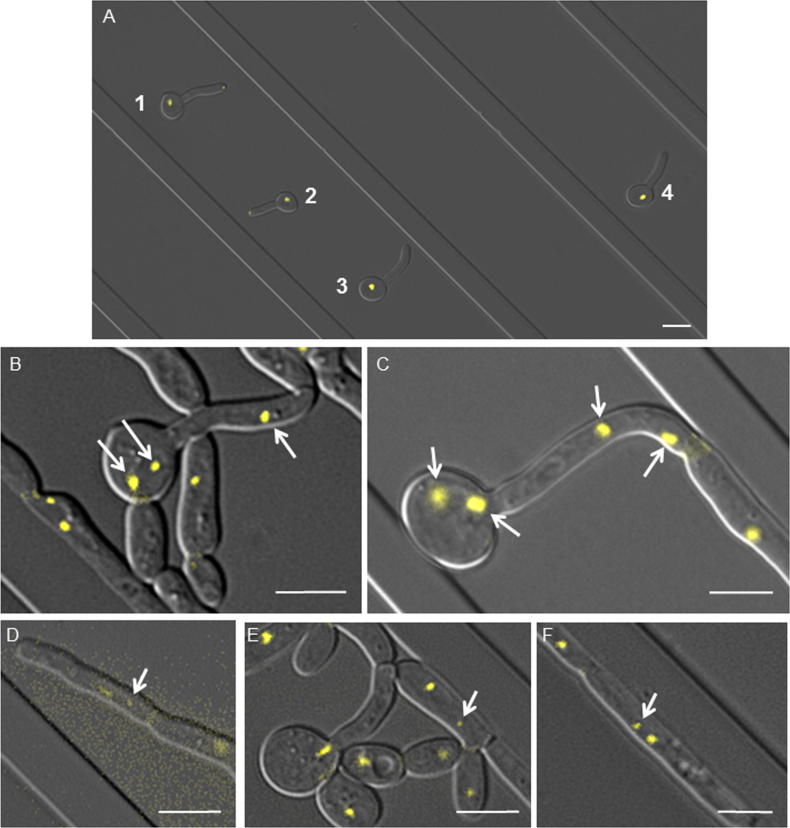
(A) Video Still Image at *t* = 0 min indicating cells numbered 1–4 from left to right. Bar = 5 μm. (B and C) Multiple full-size Nop1-YFP aggregates were maintained in some cells. Panel B is derived from Cell 1 (*t* = 434 min) in the movie. Panel C is derived from a movie made in parallel using the same strain. Bar = 5 μm. (D, E and F) Small, transient Nop1-YFP nucleolar fragments (arrows) in cells originating from (from l to *r*) Mother Yeast Cell 1 (*t* = 397 min), Cell 3 (*t* = 393 min) and Cell 4 (*t* = 388 min). Bar = 5 μm.

Nuclear division, detected with Nop1-YFP, occurred within the compartment bounded by a mature, closed septum at one end and a presumptum, which appeared prior to nuclear division, at the other end. The movie revealed a previously unrecognized degree of nuclear mobility during growth. Following mitosis, one sister nucleus stayed within the mother cell while the other crossed the presumptum into the daughter compartment. Although the nucleus in dividing mother cells was highly mobile, of the 42 nuclear divisions in the movie, none was observed to occur across the plane of the septum. Instead, a new sister nucleus often traveled some distance along the hypha before reaching the presumptum. In this and other movies, we observed that some cells generated and maintained up to 4 full-size Nop1-YFP spots, suggesting that these cells may be tetraploid, multinucleate and/or aneuploid and may arise more frequently than previously thought (Fig. 2B and C).

### 2.3 Nuclear translocation and inheritance

Prior to nuclear division in hyphal compartments, the nucleus was maintained centrally due to the positioning of vacuoles on either side of it. Post-mitotic nuclei often encountered delays in movement as they transited the presumptum, suggesting that the aperture is partially constricted compared to main body of the hyphal cell or that interaction of the nucleus with microtubules requires reorganization in order to move it beyond the septin ring. In elongating hyphae, the proximal sister nucleus remained in the mother compartment while the distal sister migrated into the daughter compartment. In some branching hyphae it was the proximal sister nucleus that migrated into the new branch (see compartments formed from Yeast Cell 1).

### 2.4 Formation of rDNA circles

On several occasions, a small, discrete spot of Nop1-YFP was spatially separated from the nucleus and remained visible for 10–23 min (Fig. 2D–F). Two of these small Nop1-YFP spots were observed in mother hyphal compartments cells and one in a daughter compartment. These could represent autonomously replicating circular or linear rDNA plasmids, which have been isolated from actively-growing *C. albicans* cells (Huber and Rustchenko, 2000). Nop1 is involved in pre-rRNA modification within the nucleolus (Tollervey et al., 1991) and thus is associated with the tandem repeats of rDNA found on ChrXII in *Saccharomyces cerevisiae* and on ChrR in *C.* *albicans*. Recombination of tandem rDNA repeats can give rise to extrachromosomal circular molecules that have the ability to replicate autonomously. We suggest that nucleolar proteins, including Nop1, likely remain associated with rDNA in these extrachromosomal plasmids, producing small nucleolar regions in addition to the major nucleolus associated with ChrR. In *S.* *cerevisiae*, nucleolar fragmentation is more frequently detected in aging cells (Sinclair and Guarente, 1997). This does not seem to be the case in *C.* *albicans* hyphae, where Nop1-YFP spots were observed in hyphal compartments during elongation but not in the older mother yeast cell, even though yeast nuclei continued to divide to produce new daughters. To our knowledge, this is the first demonstration of independent localization of small nucleolar fragments in *C.* *albicans.*

### 2.5 Simultaneous generation of hyphae and pseudohyphae

The movie provides further evidence that both hyphae and pseudohyphae can emanate simultaneously or sequentially from the same parent cell. The germ tube from Yeast Cell 1 (far left) initiated a hyphal branch but then formed a new pseudohyphal cell from the opposite side of the mother hypha. Similarly, from Yeast Cell 3, a branch elongated from the mother compartment while a pseudohypha, in which the septum formed at the mother-bud neck, developed simultaneously from the same compartment. Older cells that were proximal to the initial mother cell produced branches prior to more distal cells (Veses et al., 2009). Of the 11 branching events shown in this movie, 3 occurred adjacent to a septation site. In the remaining 8 branches, the mean distance (±SD) of branching was 4.7 ± 3.2 μm from a septation site, with an overall bias toward the apical septum, The site of branching is thought to be determined by septin positioning. In *Ashbya gossypii* (phylum Saccharomycotina, which includes *C. albicans*) branching occurs within 10 μm of the apical septum (deMay et al., 2009), while in *A. nidulans* (phylum Pezizomycotina) branches appear to form adjacent to dividing nuclei (Dynessen and Nieldsen, 2003). Our findings are therefore consistent with other fungi of the Saccharomycotina phylum.

## 3 Summary

Improved imaging methods and microfluidic formats facilitated the production of high-frame-rate movies that revealed the dynamic mobility and potential formation of aneuploids, tetraploids and/or multinucleate wild-type *C. albicans* cells under standard hyphal growth conditions. We observed that nuclei do not divide across the septal plane in hyphal cells and we could identify and track individual sister nuclei during migration after division. Finally, we observed the transient formation of nucleolar fragments, which may represent the first visualization of the formation of extrachromosomal rDNA circles in *C.* *albicans*.

## 4 Methods

The strains used in this study were *C. albicans* 8860 (Nop1-YFP/Cdc3-GFP) (Finley and Berman, 2005) and YMG7139 (Mlc1-YFP) (Crampin et al., 2005). Cells were grown in 2% glucose and 20% fetal calf serum at 37 °C to induce filamentation. Growth was observed in a micro-fabricated chamber cast in poly-dimethylsiloxane (Sylgard-184, Down Corning) using a Zeiss AxioObserver X1 running Axiovision software (v 4.8,Carl Zeiss, Germany). Images were captured every 1 min using a 16-bit CoolSNAP H^2^ cooled camera (Photometrics, USA) with a 1 s exposure for GFP and a EC Plan Neofluor 40× oil-immersion objective (NA 1.3) and a Zeiss #62 HE filter to collect both GFP and YFP emission signals simultaneously.
