# Supplementary figures and images for: High frame-rate resolution of cell division during Candida albicans filamentation
Source: Fungal Genet Biol. 2016 Mar;88:54–8. doi: 10.1016/j.fgb.2016.02.001 (PMC4767323; doi:10.1016/j.fgb.2016.02.001)

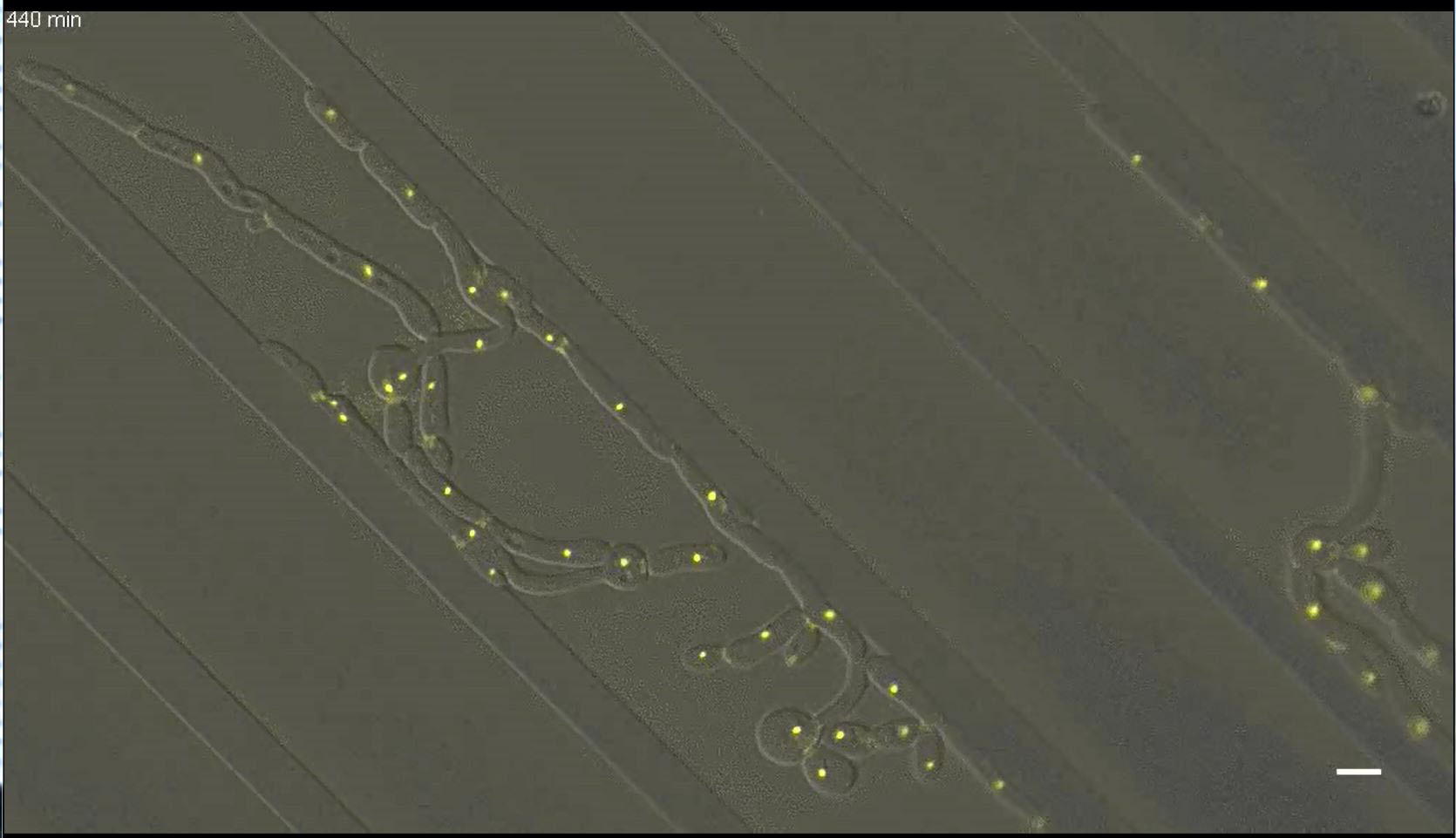

Supplement: Supplementary video 1 [file mmc1.jpg]
